# New Insights Into the Intricacies of Proneural Gene Regulation in the Embryonic and Adult Cerebral Cortex

**DOI:** 10.3389/fnmol.2021.642016

**Published:** 2021-02-15

**Authors:** Ana-Maria Oproescu, Sisu Han, Carol Schuurmans

**Affiliations:** ^1^Sunnybrook Research Institute, Biological Sciences Platform, Toronto, ON, Canada; ^2^Department of Laboratory Medicine and Pathobiology, University of Toronto, Toronto, ON, Canada; ^3^Department of Biochemistry, University of Toronto, Toronto, ON, Canada

**Keywords:** *Neurog1*, *Neurog2*, *Ascl1*, phosphorylation, protein–protein interactions, protein stability, epigenetic control, translational control

## Abstract

Historically, the mammalian brain was thought to lack stem cells as no new neurons were found to be made in adulthood. That dogma changed ∼25 years ago with the identification of neural stem cells (NSCs) in the adult rodent forebrain. However, unlike rapidly self-renewing mature tissues (e.g., blood, intestinal crypts, skin), the majority of adult NSCs are quiescent, and those that become ‘activated’ are restricted to a few neurogenic zones that repopulate specific brain regions. Conversely, embryonic NSCs are actively proliferating and neurogenic. Investigations into the molecular control of the quiescence-to-proliferation-to-differentiation continuum in the embryonic and adult brain have identified proneural genes encoding basic-helix-loop-helix (bHLH) transcription factors (TFs) as critical regulators. These bHLH TFs initiate genetic programs that remove NSCs from quiescence and drive daughter neural progenitor cells (NPCs) to differentiate into specific neural cell subtypes, thereby contributing to the enormous cellular diversity of the adult brain. However, new insights have revealed that proneural gene activities are context-dependent and tightly regulated. Here we review how proneural bHLH TFs are regulated, with a focus on the murine cerebral cortex, drawing parallels where appropriate to other organisms and neural tissues. We discuss upstream regulatory events, post-translational modifications (phosphorylation, ubiquitinylation), protein–protein interactions, epigenetic and metabolic mechanisms that govern bHLH TF expression, stability, localization, and consequent transactivation of downstream target genes. These tight regulatory controls help to explain paradoxical findings of changes to bHLH activity in different cellular contexts.

## Introduction

One-hundred years ago, Santiago Ramon y Cajal likened the pyramidal cells of the cerebral cortex to “a garden filled with innumerable trees…which can multiply their branches thanks to intelligent cultivation, send their roots deeper, and produce more exquisite flowers and fruits every day” (Jones, 1994). In his 1942 book Man on His Nature, neurophysiologist Sir Charles Sherrington compared the electrical activity of the cortex to “an enchanted loom” (Sherrington, 2009). The field of neuroscience is ripe with such metaphors that remark upon the exquisite architecture and cellular diversity of the cerebral cortex. It follows, then, that one of the most dominant inquiries in developmental neuroscience has been how this enormous cellular diversity is established and choreographed during brain development. Detangling this great mystery has important implications for our understanding of neurological disorders and diseases, as well as for the future design of therapeutic strategies to replace lost/dysfunctional neural cells.

This review centers on the regulatory events that govern proneural gene function in the developing and adult cerebral cortex. To provide a contextual framework, we first provide a high-level overview, not meant to be comprehensive, of the cellular context in which these genes function. The cerebral cortex, which is the seat of higher order cognitive functioning and sensory processing, is comprised of a six-layered neocortex and several three- or four-layered allocortical territories, including the hippocampal formation and paleocortex. Cortical territories are found in all mammals, but display enormous structural diversity across species, transitioning during evolution between smooth (lissencephalic) structures in smaller mammals such as rodents, to highly folded (gyrencephalic) structures in most extant primates and larger mammals (Lewitus et al., 2014). These gross structural differences arise due to species-specific differences in the regulatory events that control self-renewal, proliferation, mode of division (symmetric, asymmetric) and differentiation properties of neural stem cells (NSCs) and their daughter neural progenitor cells (NPCs). Distinguishing features of NSCs include maintenance into adulthood, the capacity to self-renew, and multipotency, which refers to their tri-lineage potential, or the capacity to give rise to neurons, astrocytes and oligodendrocytes. Conversely, NPCs do not self-renew, are more restricted in their proliferative potential, and may have reduced developmental potential as they acquire lineage biases.

To understand how the cerebral cortex acquires species-specific forms, it is essential to elucidate how NSC/NPC (hereafter NPC for simplicity) fate decisions are controlled. Proneural genes, which encode basic-helix-loop-helix (bHLH) transcription factors (TFs), are critical pieces to the puzzle as they control NPC decisions to divide or differentiate while also specifying neural subtype identities (Bertrand et al., 2002; Wilkinson et al., 2013; Guillemot and Hassan, 2017; Dennis et al., 2019). At face value, the functions of proneural genes appear simplistic, but their activities are tightly regulated by both cell intrinsic and extrinsic influences. Here we review the regulatory mechanisms that govern proneural gene function in embryonic and adult cortical domains, drawing parallels to other bHLH genes, brain regions, non-neural tissues, and non-mammalian species when comparison is informative. Of note, unless otherwise specified, the animal work cited was conducted using murine transgenic models, tissues or cells.

### Introduction to Proneural Genes

Proneural genes encode type II, tissue-specific bHLH TFs that are expressed in the nervous system and have evolutionarily conserved roles in promoting neural cell fate specification and differentiation (Bertrand et al., 2002; Wilkinson et al., 2013; Guillemot and Hassan, 2017; Dennis et al., 2019). Proneural genes were first identified and characterized in *Drosophila melanogaster* where they belong to two main families that each specify distinct neural cell fates: *achaete-scute* complex (AS-C) and *atonal-*related genes (Bertrand et al., 2002; Huang et al., 2014). In the fly, bHLH genes that are defined as proneural are expressed in uncommitted ectodermal precursors and have the ability to: (1) select single ectodermal precursors within a proneural cluster to become neural by activating Notch/Delta-mediated lateral inhibition, and (2) specify neural precursor identity by activating generic and subtype-specific neuronal differentiation genes. While vertebrate and invertebrate proneural genes share several features, a major difference is that in vertebrates, proneural gene expression initiates in NPCs that are already specified as neural. With this difference in mind, vertebrate bHLH genes are defined as proneural if they: (1) are expressed in dividing NPCs, usually those at the apex of lineage hierarchies, (2) drive NPCs to differentiate into neuronal or glial cells, (3) specify neural subtype identities, and (4) activate Notch signaling in neighboring NPCs by inducing the expression of Notch ligands, such as *Dll1* and *Dll3* (Bertrand et al., 2002; Wilkinson et al., 2013; Guillemot and Hassan, 2017; Dennis et al., 2019). Based on these criteria, four proneural genes are expressed in the developing and/or adult cerebral cortex: *Neurogenin (Neurog) 1, Neurog2*, *Neurod4* (aka *Math3*), and *Achaete-scute family bHLH transcription factor 1* (*Ascl1;* aka *Mash1*) (Bertrand et al., 2002; Wilkinson et al., 2013; Guillemot and Hassan, 2017; Dennis et al., 2019). All other commonly studied neural bHLH genes, such as *Neurod1, Neurod2, Neurod6* and others are instead properly termed ‘neuronal differentiation’ genes because of their later expression/function in neural lineages, either in later-stage progenitors with a restricted proliferative and differentiation potential [e.g., *Neurod1* (Pleasure et al., 2000)], and/or in postmitotic neurons [e.g., *Neurod2, Neurod6* (Bormuth et al., 2013; Guzelsoy et al., 2019)]. In this review we mainly focus on the cortical functions of *Neurog1, Neurog2* and *Ascl1*, which have been most extensively studied.

To bind DNA, proneural bHLH TFs must dimerize, either with other proneural TFs or with type I bHLH factors, also known as E-proteins (Murre et al., 1989). E-proteins, which have more ubiquitous expression patterns than class II bHLH TFs, are encoded by three genes: *Tcf4* (aka *E2-2*), *Tcf12* (aka *HEB*), and *Tcf3* (aka *E2A*), the latter encoding E12 and E47 splice variants (Bertrand et al., 2002; Wang and Baker, 2015). Proneural TFs can also dimerize with HLH proteins of the Id (inhibitor of DNA-binding) family, which lack the basic DNA-binding domain and thus form non-functional heterodimers (Wang and Baker, 2015). To activate transcription, bHLH dimers bind to Ephrussi-box (E-box) sequences (CANNTG) in regulatory regions of the genome (Murre et al., 1989; Wang and Baker, 2015). ChIP-seq analyses have revealed that different proneural TF binding sites have differential enrichment of the central two E-box residues; Neurog2 favors CAKMTG motifs (K: G/T nucleotides, M: A/C nucleotides), with the CAGATG motif predominant, while Ascl1 preferentially binds sites with CAGSTG motifs (S: G/C nucleotides), with the CAGCTG motif predominant (Wapinski et al., 2013; Raposo et al., 2015; Aydin et al., 2019). Despite these known biases, the binding of proneural TF hetero- or homo-dimers to their cognate sites is highly context-specific and tightly regulated, which is the subject of this review.

### A Primer on Neocortical Development

To set the stage for the embryonic context in which proneural TFs function, we briefly outline critical developmental transitions. The neurons and macroglial cells (oligodendrocytes, astrocytes) that make up the adult cerebral cortex are derived from multipotent NPCs located in the dorsal telencephalon (cortex), with some additional contributions from ventral telencephalic (subcortical) NPCs. Telencephalic NPCs are parcellated into apical and basal compartments (Taverna et al., 2014). Apical NPCs reside in the ventricular zone (VZ), a single cell-layered neurepithelium that appears pseudostratified due to interkinetic nuclear migration, with G2/M-phase nuclei moving to the apical surface whereas S-phase nuclei move basally (Taverna et al., 2014). Apical NPCs are termed neuroepithelial cells (NECs) prior to neurogenesis and initially divide symmetrically to expand the NPC pool (Gotz and Huttner, 2005). When neurogenesis begins, at approximately embryonic day (E) 11 in mouse, NECs transform into apical radial glia (aRG), which remain in the VZ, but switch to self-renewing asymmetric neurogenic divisions to give rise to one aRG and either one new neuron (direct neurogenesis) or one basal progenitor (indirect neurogenesis) (Gotz and Huttner, 2005; Bultje et al., 2009). aRG and NECs differ at the transcriptomic level, with aRGs initiating the expression of several glial markers (Taverna et al., 2014). In rodents, basal progenitors, which form a subventricular zone (SVZ), predominantly include neuronal-committed intermediate progenitor cells (INPs) that have a limited proliferative capacity (1–2 divisions) and undergo terminal symmetric neurogenic divisions (Haubensak et al., 2004; Miyata et al., 2004; Noctor et al., 2004; Englund et al., 2005; Kowalczyk et al., 2009; Taverna et al., 2014). Further stratifications of these apical and basal NPC pools have been made based on morphological and gene expression criteria and are reviewed elsewhere (Taverna et al., 2014).

Cortical NPCs give rise in a sequential fashion to excitatory glutamatergic neurons that form the six layers of the cortical plate between E11-E17 in mouse (Caviness, 1982; Caviness et al., 1995; Takahashi et al., 1999), followed by astrocytes, beginning at E16 (Bayraktar et al., 2014), and then oligodendrocytes, beginning early postnatally (Kessaris et al., 2006). The earliest-born cortical neurons form a preplate that is later split into an overlying marginal zone (layer I) and an underlying subplate (layer VII), the latter a transient neuronal layer that nevertheless plays important roles in thalamocortical axonal pathfinding and in guiding neuronal migration (Ohtaka-Maruyama, 2020). Layer VI corticothalamic neurons are born next, followed by the sequential differentiation of layer V subcerebral and callosal neurons, layer IV internal granular layer neurons, and finally, layer II/III corticocortical neurons, two layers that are fused in mouse (Caviness, 1982; Kast and Levitt, 2019). GABAergic interneurons and oligodendrocytes also populate cortical domains, but they are born in the ventral telencephalic (subcortical) VZ/SVZ and enter the cortex via tangential migration (Peyre et al., 2015).

The progressive nature of laminar fate determination raises the question of how cortical NPCs change over time (Pearson and Doe, 2004). Seminal studies involving heterochronic transplantation experiments in ferrets revealed that early-stage cortical NPCs are multipotent, responding to new environmental signals to generate alternative laminar identities post-transplant, but only when in S-phase of the cell cycle, whereas later stage cortical NPCs lose their ability to respond to early environmental signals (McConnell and Kaznowski, 1991; Frantz and McConnell, 1996; Bohner et al., 1997; Desai and McConnell, 2000). These findings were corroborated by retroviral lineage tracing experiments, which confirmed that *early* cortical NPCs are multipotent and give rise to neuronal clones that span cortical layers, whereas *late* NPCs are fate restricted and only generate upper layer neurons (Luskin et al., 1988; Price and Thurlow, 1988; Walsh and Cepko, 1988). More recently, genetic lineage tracing experiments using various Cre drivers (Franco et al., 2012; Guo et al., 2013; Eckler et al., 2015) and Mosaic Analysis with Double Markers (MADM) (Gao et al., 2014) have confirmed that cortical NPCs are multipotent at the population and clonal level, although some fate-restricted NPCs may also exist (Franco et al., 2012; Gil-Sanz et al., 2015). How NPCs give rise to such diverse neural cell types in a stereotypically defined manner has been the subject of study for several decades now (Pearson and Doe, 2004). The importance of intrinsic factors was demonstrated by plating cortical NPCs at clonal density, which generated stereotyped lineage trees that matched those seen *in vivo* (Qian et al., 2000). Since then, revolutionary new technologies such as FlashTag and single cell (sc) RNA-seq have identified sequential transcriptional waves that successively define apical and basal NPCs and daughter neurons (Telley et al., 2016). Further studies with these techniques identified two axes of NPC transcriptional organization throughout the neurogenic period: a “birthdate axis” in which the transcriptional state varies depending on embryonic age, and a “differentiation axis,” which drives NPCs to differentiate in a conserved sequence regardless of neuronal birthdate (Telley et al., 2019). Interestingly, this work showed that late-stage apical NPCs (E14/E15) have predominantly environment-sensing transcriptional properties, with activation of genetic programs related to ion transport and cell-cell or cell-matrix interaction-related processes, as opposed to the cell-intrinsic transcriptional programs in earlier apical NPCs (Telley et al., 2019).

From these pioneering studies of cortical NPCs, *Neurog2* was highlighted as a critical ‘neurogenic’ (actually, proneural) gene as it is expressed at high levels in apical and basal NPCs and at low levels in newborn neurons (Telley et al., 2016), consistent with earlier immunostaining studies (Hand et al., 2005). Functional assays demonstrating that *Neurog1* and *Neurog2* are true proneural genes pre-dated these studies by a decade or more and involved classical loss- and gain-of-function assays (Fode et al., 2000; Parras et al., 2002; Schuurmans et al., 2004; Mattar et al., 2008; Dixit et al., 2011, 2014; Kovach et al., 2013; Han et al., 2018). From these studies, *Neurog2* and *Neurog1* were shown to be necessary and sufficient to specify the excitatory, glutamatergic neuronal identity of early-born (layer V, VI) cortical neurons, as well as Cajal-Retzius neurons, which populate layer I (Dixit et al., 2014). In contrast, *Ascl1*, which is expressed at the highest levels in subcortical NPCs, is necessary and sufficient to specify a GABAergic neuronal or oligodendrocyte fate in the ventral telencephalon (Casarosa et al., 1999; Horton et al., 1999; Schuurmans et al., 2004; Parras et al., 2007). Interestingly, *Ascl1* is also expressed at lower levels in cortical NPCs (Britz et al., 2006), where it also biases NPCs toward an oligodendrocyte fate (Han et al., 2020). In addition, *Ascl1* is also required for the generation of a subset of glutamatergic Cajal-Retzius neurons, as opposed to the GABAergic fates specified by this TF in ventral telencephalic domains, highlighting the importance of cell context in dictating how these proneural genes function (Dixit et al., 2011). Finally, the transient expression of *Neurog2* and *Ascl1* in newborn neurons also has functional consequences, as these genes play a role in guiding neuronal migration by regulating expression of the Rho GTPases, *Rnd2* and *Rnd3*, respectively (Heng et al., 2008; Pacary et al., 2011, 2013).

### Lissencephalic Versus Gyrencephalic Cortical Development

**FIGURE 1:**
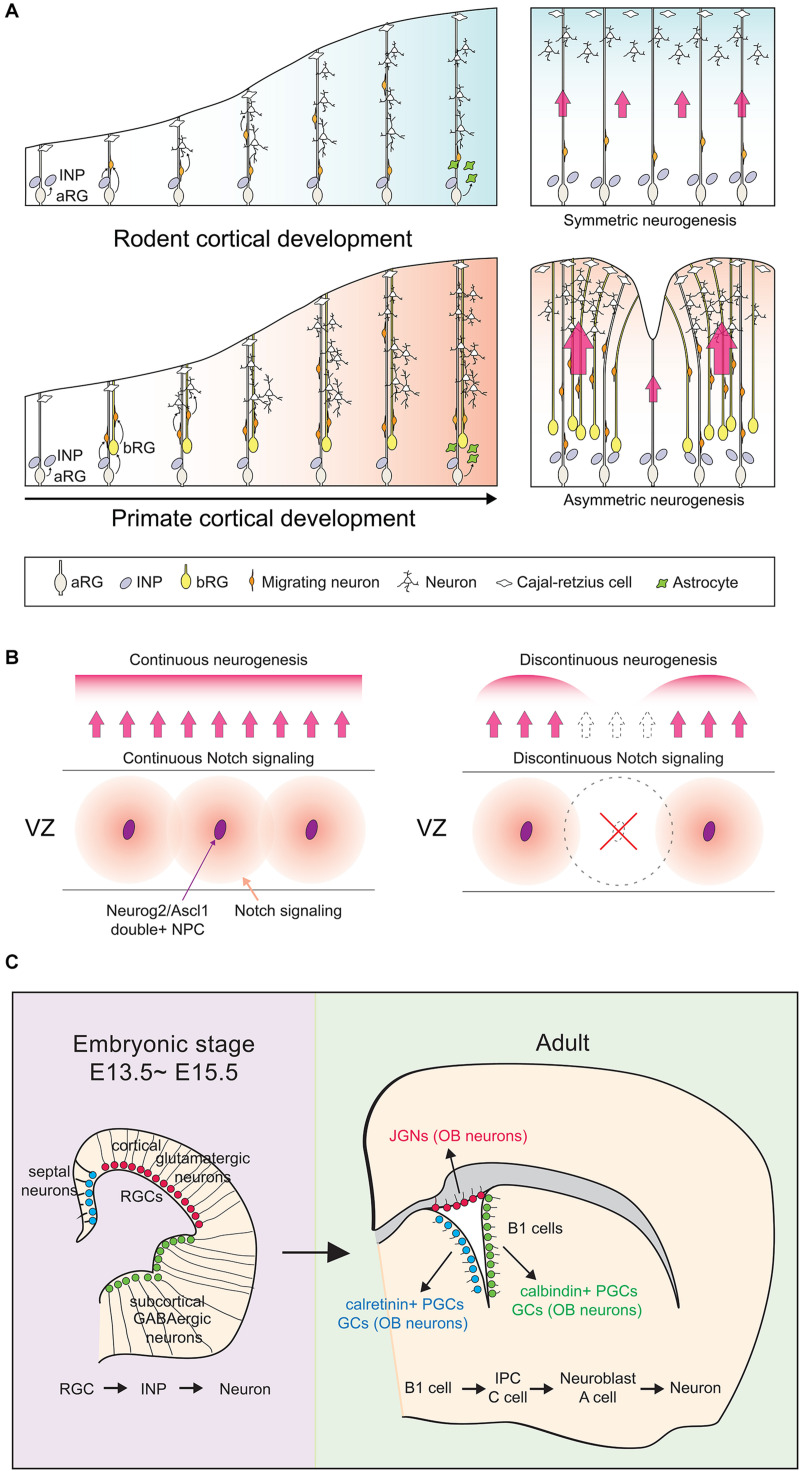
Cortical development between species and developmental stages. **(A)** In both lissencephalic and gyrencephalic species, apical radial glial (aRG) cells can divide asymmetrically to give rise to another aRG and either a nascent neuron (direct neurogenesis) or a neuronal-committed intermediate neuronal progenitor (INP; indirect neurogenesis). Gyrencephalic species like primates have an additional population of radial glial cells, the basal RG (bRG), which contribute more dividing INP cells to in turn generate more upper layer neurons in primate cortices. In the panels on the right, red arrows signify migratory routes of nascent neurons traveling along aRG processes. Larger arrows signify cortical regions with increased neurogenesis. **(B)** *Neurog2/Ascl1* double-positive NPCs (purple ovals) act as ‘niche’ cells in the ventricular zone (VZ) of the rodent cortex, preventing the formation of cortical folds by maintaining continuous Notch signaling patterns. **(C)** Unlike the majority of actively dividing apical radial glia in E13.5–15.5 cortices, a subset remains quiescent and is set aside to become adult neural stem cells, the ‘B1’ cells. B1 cells reside in the adult cortical VZ and retain their regional identities. For adult B1 cells to exit quiescence and become neurogenic, upregulation of a cortical proneural gene like *Neurog2* or *Ascl1* must occur. aRG, apical radial glia; INP, intermediate neuronal progenitor; bRG, basal radial glia; JGN, juxtaglomerular neuron; OB, olfactory bulb; PGC, periglomerular cell; GC, granule cell.

Studies of non-human primates (NHP) and human cortices have revealed that the apical NPC pool has expanded to include both aRG and outer or basal RG (bRG), the latter forming a large outer SVZ (oSVZ) not present in rodents (Lukaszewicz et al., 2005; Zecevic et al., 2005; Bayatti et al., 2008; Martinez-Cerdeno et al., 2012; Dehay et al., 2015). Like aRG, bRG are self-renewing and generate neurons by giving rise to transit-amplifying INPs (Fietz et al., 2010; Hansen et al., 2010; Reillo et al., 2011). However, INPs divide several more times in gyrencephalic species than in rodents to generate more later-born, upper-layer or supragranular neurons that make primate cortices larger with many folds (Molnar et al., 2011; Falk and Hofman, 2012; Stahl et al., 2013; Pollen et al., 2015) (Figure 1A). Several genes that promote basal NPC expansion can induce cortical folding in lissencephalic mammals, such as mice, or alter folding in gyrencephalic species, such as ferrets (Hansen et al., 2010; Fietz et al., 2012; Stahl et al., 2013; de Juan Romero et al., 2015; Florio et al., 2015, 2018; Ju et al., 2016; Wang et al., 2016; Fiddes et al., 2018; Suzuki et al., 2018; Chizhikov et al., 2019). Conversion from a lissencephalic to gyrencephalic cortex is also associated with alterations of the simple radial trajectories of migrating neurons in lissencephalic species, to more circuitous, tangential routes in gyrencephalic species (Del Toro et al., 2017; Llinares-Benadero and Borrell, 2019). Recent studies have revealed an unexpected role for *Neurog2* and *Ascl1* co-expression in sustaining a lissencephalic form in the rodent cortex due to the essential role that double^+^ NPCs play in patterning Notch signaling, which impacts the symmetry of radial glial trajectories (Han et al., 2020).

### Embryonic Versus Adult Neurogenesis

While NSCs persist into adulthood, they differ from embryonic NSCs in several ways. Firstly, the adult NSC transcriptional profile is more closely related to astrocytes than to embryonic NSCs (Beckervordersandforth et al., 2010). Secondly, most embryonic NSCs are actively dividing and neurogenic, whereas adult NSCs are mainly quiescent and gliogenic (Gotz et al., 2016). Indeed, up to 90% of adult NSCs are quiescent in the adult brain at any given time, with cell cycle times ranging from 1 day to 3 months (Ponti et al., 2013; Reeve et al., 2017). Adult NSCs that become ‘activated’ are also restricted to a few neurogenic zones and repopulate only specific brain regions. For instance, the ventricular-subventricular zone (V-SVZ) repopulates the murine olfactory bulb and human striatum, while the subgranular zone (SGZ) repopulates the mouse/human dentate gyrus (Spalding et al., 2013; Ernst et al., 2014; Urban and Guillemot, 2014; Gotz et al., 2016; Boldrini et al., 2018; Ruddy and Morshead, 2018; Sorrells et al., 2018). Outside of these niches, the adult NSC response is limited.

Conversely, most embryonic NSCs divide rapidly *in vivo*, with cell cycle times of 8–18 h (Takahashi et al., 1995). However, a small but important pool of embryonic NSCs is slow-dividing; these are the embryonic precursors of adult NSCs, the origins of which had remained elusive until recently (Furutachi et al., 2013, 2015; Fuentealba et al., 2015). Using barcoding, a genetic lineage tracing method that can identify clonal relationships between widely distributed cells, it was revealed that a subset of E13.5-E15.5 aRG, termed ‘pre-B1 cells,’ are set aside as slow-dividing NPCs that will later become adult B1 cells (Fuentealba et al., 2015) (Figure 1B). B1 cells are adult NSCs, which when activated give rise to transit amplifying intermediate precursor cells (IPCs, C cells) that generate neuroblasts (A cells) that migrate through the rostral migratory stream (RMS) to the olfactory bulb (Li and Clevers, 2010). B1 cells retain regional identities; dorsal NSCs give rise to glutamatergic juxtaglomerular neurons (JGNs), ventral NSCs to calbindin^+^ periglomerular cells (PGCs) and granule cells (GCs), and septal NSCs to calretinin^+^ PGCs and GCs (Brill et al., 2009; Fuentealba et al., 2015). With respect to the focus of this review, for adult NSCs to become activated and neurogenic, neural determinants such as *Neurog2* and *Ascl1*, which are expressed at high levels in embryonic NSCs and low levels in adult NSCs, must be upregulated (Gotz et al., 2016; Guillemot and Hassan, 2017). We discuss the associated regulatory mechanisms herein.

## Intersection Between Proneural Genes and Extracellular Signaling Pathways

### Notch Signaling Controls Proneural Gene Expression and Oscillations

*Neurog2* and *Ascl1* are classical proneural genes, rapidly inducing NPC cell cycle exit and differentiation when misexpressed in the embryonic cortex (Britz et al., 2006; Mattar et al., 2008; Kovach et al., 2013; Wilkinson et al., 2013). Yet curiously, *Neurog2* (Hagey and Muhr, 2014) and *Ascl1* (Castro et al., 2011; Li et al., 2014) can also induce proliferation when expressed in some cellular contexts. Moreover, during normal development, *Neurog1, Neurog2* and *Ascl1* are mainly expressed in dividing NPCs (Britz et al., 2006). These findings raise the question of how proneural gene expression is compatible with both pro-proliferative and pro-differentiative NPC phenotypes. This conundrum was partially resolved in ground-breaking studies that demonstrated that *Neurog2* and *Ascl1* are expressed in 2–3 hr oscillatory cycles in dividing NPCs versus at sustained levels in NPCs that differentiate (Shimojo et al., 2008; Imayoshi et al., 2013; Ochi et al., 2020).

**FIGURE 2:**
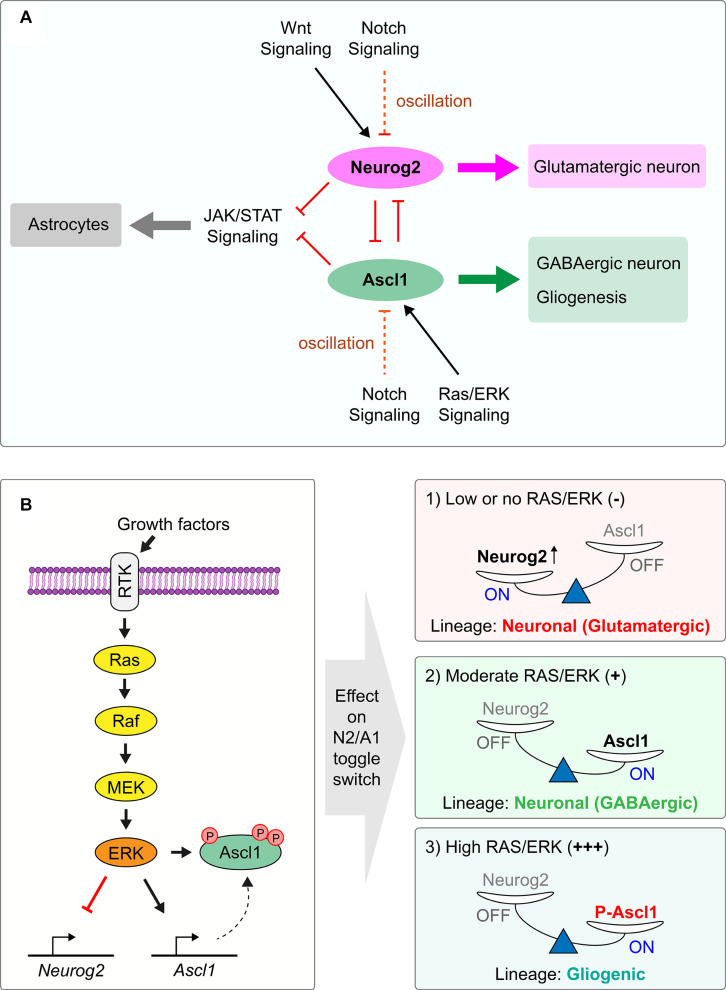
Regulation of the *Neurog2/Ascl1* proneural gene toggle switch by extracellular signaling pathways. **(A)** Proneural TFs *Neurog2* and *Ascl1* are competing lineage determinants in the cortex, specifying glutamatergic pyramidal neurons and GABAergic interneurons, respectively. *Neurog2* and *Ascl1* have cross-repressive interactions with each other and form a bistable toggle switch, preventing lineage commitment in double-positive NPCs. Environmental signals regulate the expression of each gene to turn on the expression of one proneural TF and turn off the other. Notch signaling controls expression of both *Neurog2* and *Ascl1* through lateral inhibition, with Hes1 protein driving the 2–3 h transcriptional oscillatory cycles of the proneural genes. Extracellular Wnt promotes *Neurog2* and suppresses *Ascl1* expression, acting in early neurogenesis. Conversely, Ras/ERK signaling favors *Ascl1* over *Neurog2* expression. **(B)** Ras/ERK signaling cascade activation is achieved by ligand binding to RTK, culminating in the phosphorylation of Ascl1 protein and activation of Ascl1 expression, tipping the Neurog2/Ascl1 toggle switch in favor of Ascl1. At moderate Ras/ERK activation, this leads to GABAergic neuronal specification by Ascl1, while at higher levels of Ras/ERK activation, this leads to gliogenic specification by phosphorylated Ascl1.

Notch signaling is the driving force behind oscillatory proneural gene expression (Kageyama et al., 2008, 2020) (Figure 2). In a process known as ‘lateral inhibition,’ NPCs that express high levels of the proneural TFs transactivate the expression of cell-membrane tethered Notch ligands such as *Dll1* and *Dll3* (Castro et al., 2006; Henke et al., 2009), which bind Notch receptors on neighboring NPCs. Upon ligand binding, Notch is proteolytically cleaved to form a Notch intracellular domain (NICD) that translocates to the nucleus where it binds to Rbpj, a DNA binding protein. NICD-Rbpj complexes transcribe downstream genes, including *hairy and enhancer of split* (*Hes*) *1* and *Hes5*, which encode bHLH transcriptional repressors that recruit Groucho/TLE co-repressors and bind to N-boxes (CACNAG), directly repressing proneural gene transcription to form a lateral inhibitory loop (Kageyama et al., 2007, 2008, 2020; Kovach et al., 2012; Huang et al., 2014). *Hes1* is also expressed in 2–3 h oscillatory cycles, and Hes1 protein drives its dynamic expression through direct repression of its own transcription, as well as indirectly driving oscillatory expression of the proneural genes through transcriptional repression (Shimojo et al., 2008, 2011). Consequently, *Hes* and proneural genes are expressed out-of-phase with one another in ‘salt-and-pepper’ expression profiles, referring to their scattered expression when captured at individual time points (Kageyama et al., 2008). Notably, while these oscillatory cycles are transcriptionally driven, proneural proteins also oscillate as they have short intracellular half-lives (< 30 min) and are rapidly degraded with each transcriptional cycle (Nguyen et al., 2006; Kovach et al., 2013; Urban et al., 2016).

While sustained proneural TF expression biases NPCs toward differentiation, it can also maintain the NPC pool by allowing neighboring NPCs with activated Notch signaling to continue to proliferate. *Hes1/5-*mediated repression of proneural genes is essential to maintain the NPC pool, with co-deletion of *Hes1/5* or *Rbpj*, their upstream regulator (Son et al., 2020), leading to precocious neurogenesis and NPC pool depletion (Ohtsuka et al., 1999; Hatakeyama et al., 2004). Strikingly, proneural genes also regulate the patterning of Notch signaling, with NPCs that co-express *Neurog2* and *Ascl1* acting as Notch-ligand expressing niche cells, the deletion of which disrupts the continuity of Notch signaling, resulting in cortical folding (Han et al., 2020) (Figure 1B).

Notably, there is also evidence for a Notch-independent mode of Rbpj function in regulation of bHLH TFs. While Rbpj suppresses *Neurog1* transcription in NPCs, it positively regulates *Neurog1* expression in migrating postmitotic neurons independent of Notch pathway activation (Son et al., 2020). Thus, as shown for *Neurog2* (Hand et al., 2005), *Neurog1* is expressed in dividing NPCs and newborn neurons, but distinct regulatory mechanisms drive its expression in the two cell types (Son et al., 2020). Interestingly, Rbpj also binds a conserved binding motif in the *Ascl1* promoter in the locus coeruleus (Shi et al., 2012), and Rbpj directly represses *Atoh7*, another bHLH proneural gene, in a Notch-independent fashion in the retina (Miesfeld et al., 2018). Further studies are required to elucidate the extent to which Rbpj regulates *Neurog1, Neurog2*, and *Ascl1* expression through Notch-dependent and -independent modes in the embryonic cortex.

### Ras/ERK Signaling Regulates a *Neurog2-Ascl1* Toggle Switch

During embryogenesis, cortical NPCs differentiate into glutamatergic neurons and later astrocytes, but retain the potential to divert to embryonic subcortical fates (GABAergic neurons, oligodendrocytes), as revealed by the mutation of several cortical transcription factors (Theil et al., 1999; Stoykova et al., 2000; Tole et al., 2000; Muzio et al., 2002a, b; Schuurmans et al., 2004; Kroll and O’Leary, 2005), or when Ras/ERK signaling is ectopically activated (Chandran et al., 2003; Gabay et al., 2003; Hack et al., 2004; Kessaris et al., 2006; Li et al., 2014) (Figure 2). These events all induce a *Neurog2* to *Ascl1* transition and drive a dorsal-to-ventral re-specification of NPCs, indicating a lineage bifurcation point regulated by Neurog2 and Ascl1. While Neurog2 and Ascl1 both function as transcriptional activators themselves (Castro and Guillemot, 2011; Kovach et al., 2013), they are mutually transcriptionally cross-repressive; in *Neurog2* null mutants, *Ascl1* is upregulated and subcortical phenotypes are generated in the cortex (Fode et al., 2000; Schuurmans et al., 2004), while conversely, *Ascl1* can repress *Neurog2* expression when misexpressed in cortical NPCs (Han et al., 2020). *Neurog2* is also required to repress *Ascl1* expression in multipotent retinal progenitor cells (Hufnagel et al., 2010). Given that Neurog2 functions as a transcriptional activator, the mechanism for its repression of *Ascl1* transcript and protein expression is indirect, and remains to be fully elucidated. Partial features include that Neurog2 acts through a yet unknown transcriptional regulator to repress *Etv1* expression, which indirectly regulates *Ascl1* expression through repression of *Hes5*, a known transcriptional repressor of *Ascl1* (Kovach et al., 2013). Notably, proneural gene cross-repression in the cortex may be limited to competing lineage determinants such as *Neurog2* and *Ascl1* (Han et al., 2020), as *Neurog2* is instead required to positively regulate the transcription of the functionally related proneural gene, *Neurog1* (Fode et al., 2000). However, mutant analyses in the retina revealed that in the absence of *Neurog2, Ascl1* or *Neurod4*, the other two bHLH genes are upregulated (Akagi et al., 2004), indicative of cross-repressive interactions that further support context-specific functions of these genes.

Strikingly, *Neurog2* and *Ascl1* are also cross-repressive at the functional level; *Neurog2* inhibits the ability of *Ascl1* to promote a glioblast fate, while *Ascl1* inhibits the ability of *Neurog2* to specify a glutamatergic neuronal identity in cortical NPCs (Han et al., 2020) (Figure 2). This cross-repression at the protein level may be mediated by the formation of less transcriptionally active Neurog-Ascl1 heterodimers, reviewed in greater detail below. Taken together, these findings invoke comparisons to other stem cell systems in which pairs of TFs that specify different cell fates are in some instances co-expressed in the same progenitor cell, and their mutual cross-antagonism prevents fate specification and differentiation to maintain cellular bi- or multi-potency (Chickarmane et al., 2009; Dillon, 2012; Okawa et al., 2016; Brand and Morrissey, 2020). The co-expression of distinct lineage determinants has the added purpose of ‘priming’ progenitor cells for subsequent lineage selection, as downstream genes in either lineage can be readily transcribed. In the lingo of computational biologists, antagonistic TF pairs form a gene regulatory network motif known as a toggle switch (Huang et al., 2007; Chickarmane et al., 2009; Enver et al., 2009; Zandi et al., 2010; Strasser et al., 2012). Based on these operational criteria, *Neurog2* and *Ascl1* form a toggle switch to prevent lineage commitment in the embryonic cortex (Han et al., 2020).

Ras/ERK signaling is a critical regulator of the *Neurog2-Ascl1* toggle switch, and therefore it is important to understand how this signal transduction pathway is regulated in the embryonic cortex (Li et al., 2014). Ras/ERK signaling is activated by both pro-proliferative growth factors, such as epidermal growth factor (Egf) and fibroblast growth factor (Fgf) (Ghosh and Greenberg, 1995; Vaccarino et al., 1999; Raballo et al., 2000; Lukaszewicz et al., 2002; Imamura et al., 2008; Wang et al., 2009), and by pro-differentiative factors, including *platelet-derived growth factor* (*PDGF*) (Menard et al., 2002), *nerve growth factor* (*Ngf*) (Greene and Tischler, 1976; Vaudry et al., 2002), *neurotrophin 3* (*Ntf3*) (Lukaszewicz et al., 2002; Ohtsuka et al., 2009), and *brain derived neurotrophic factor* (*Bdnf*) (Barnabe-Heider and Miller, 2003; Ito et al., 2003; Medina et al., 2004; Fukumitsu et al., 2006; Bartkowska et al., 2007). Each of these signals bind receptor tyrosine kinase (RTK) receptors. The kinetics of RTK/ERK signaling is critical to its function, in that the apparently divergent effects of RTK/ERK signaling on proliferation versus differentiation are explained by the ability of Ngf/Ntrk1 to activate ERK in a sustained manner, whereas Fgf induces strong, transient ERK activation (Marshall, 1995; York et al., 1998). Mechanistic insights have also been gained into how Fgf activation biases NPCs to acquire an oligodendrocyte fate, both in the telencephalon and spinal cord (Gabay et al., 2003; Furusho et al., 2011; Li et al., 2014; Farreny et al., 2018), where Fgf acts in combination with Shh in an evolutionarily conserved manner (Esain et al., 2010). Mechanistically, downstream activation of ERK directly phosphorylates Ascl1, and higher levels of RAS/ERK activation biases this proneural TF to preferentially transactivate glioblast genes instead of promoting a GABAergic neuronal identity (Li et al., 2014).

During cortical development, activation of Ras/ERK signaling is spatially and temporally regulated, as revealed by the dynamic expression of phospho-p44/42 MAPK (Erk1/2) (Thr202/Tyr204), which is initially detected in the antihem adjacent to the lateral pallium where neurogenesis is first initiated in the cortex (Miyama et al., 1997), before spreading across the VZ by E14.5 (Li et al., 2014). Notably, the expression of pErk1/2 matches the pattern of expression of fibroblast growth factor receptor 3 (*FGFR3)* and a set of ets-domain transcription factors activated downstream of RTK signaling, including *Etv1, Etv4* and *Etv5* (Hasegawa et al., 2004; Li et al., 2014). The Etv transcription factors act as downstream effectors of FGF signaling and participate in regulating the *Neurog2-Ascl1* toggle switch; *Neurog2* indirectly represses *Etv1*, which in turn indirectly represses *Ascl1* as described above (Kovach et al., 2013). Taken together, these studies highlight the multiple points of intersection between the RAS/ERK signal transduction pathways and proneural genes.

### Wnt Signaling Promotes *Neurog2* Expression in a Temporally Defined Manner

Consistent with a role for canonical Wingless/INT (Wnt) signaling in specifying a cortical identity, two transgenic reporters for this pathway, BAT-gal (Maretto et al., 2003) and TCF-lacZ (Liu et al., 2006), are both expressed at higher levels in the dorsal versus ventral telencephalon (Backman et al., 2005; Machon et al., 2005; Li et al., 2012). Upon Wnt binding to LRP/Frizzled receptor complexes, β-catenin (encoded by *Ctnnb1*) is stabilized and translocates to the nucleus where it forms active transcriptional complexes with Tcf1. Conditional knock-out (cKO) of *Ctnnb1* in early cortical NPCs, prior to neurogenesis, downregulates *Neurog2* and upregulates *Ascl1* expression (Backman et al., 2005). Conversely, the addition of exogenous Wnts allows dissociated dorsal telencephalic chick cells or murine cortical neurospheres, which normally ventralize rapidly (Gabay et al., 2003), to maintain their dorsal identity *in vitro* (Gunhaga et al., 2003; Machon et al., 2005; Watanabe et al., 2005). Similarly, misexpression of *Ctnnb1* in subcortical NPCs induces ectopic *Neurog1/2* expression and suppresses *Ascl1* (Hirabayashi et al., 2004; Backman et al., 2005). Thus, the Wnt pathway also controls the *Neurog2-Ascl1* toggle switch, biasing NPCs toward *Neurog2* expression and a cortical cell fate (Figure 2).

Wnt reporter activity drops off dramatically in cortical NPCs in mid-neurogenesis (E15.5-E16.5), correlating with the time when *Neurog2* function is attenuated (Backman et al., 2005; Machon et al., 2005; Li et al., 2012). In the absence of Wnts, glycogen synthase kinase (GSK) is activated, forming a destruction complex with axin, APC and other molecules that phosphorylates and targets β-catenin for degradation. GSK3 also directly phosphorylates Neurog2 during mid-late corticogenesis through phosphorylation (Li et al., 2012), which promotes the formation of Neurog2-E47 heterodimers at the expense of more transcriptionally active Neurog2-Neurog2 homodimers (Li et al., 2012). Notably, Neurog2-E47 heterodimers have longer half-lives than Neurog2-Neurog2 homodimers, so their reduced transcriptional activity is not due to enhanced degradation (Li et al., 2012), but rather due to DNA binding preferences, as discussed further below. Therefore, Wnt signaling intersects Neurog2 function at a few levels, not only promoting Neurog2 expression, but also regulating its activity.

### Intersection Between Astrocytic Signals and Proneural Genes

Several signaling pathways induce cortical NPCs to differentiate into astrocytes (Stipursky et al., 2012), including: (1) cytokines, such as cardiotrophin-1 (CT-1), leukemia inhibitory factor (LIF), and ciliary neurotrophic factor (CNTF), all of which activate JAK/STAT signaling (Bonni et al., 1997; Koblar et al., 1998; Barnabe-Heider et al., 2005; He et al., 2005); (2) bone morphogenetic proteins (BMPs) (Bonni et al., 1997) and transforming growth factor beta (Tgfb), which function through downstream Smad effector proteins to promote astrocyte maturation (Gross et al., 1996; Bonaguidi et al., 2005); and (3) Notch-Delta signaling, as described above (Gaiano et al., 2000; Ge et al., 2002; Grandbarbe et al., 2003; Kamakura et al., 2004; Wu et al., 2017).

Interestingly, cortical NPCs expressing *Neurog1/2* and/or *Ascl1* are biased against an astrocytic fate (Han et al., 2020) (Figure 2), with several mechanisms of action identified. Firstly, Neurog1, which declines in expression when astrocyte differentiation begins at E15.5 (He et al., 2005; Han et al., 2018), sequesters transcriptional co-activators (CBP/p300) away from Stat1/3 and Smad1 TFs, preventing the transactivation of downstream astrocytic genes such as GFAP by cytokine and BMP/Tgfb signaling (Sun et al., 2001). Secondly, Neurog1 induces the transcription of miR-9, which downregulates the expression of genes in the JAK/STAT pathway (Zhao et al., 2015). Conversely, signaling pathways promoting astrocytic fate impair the ability of proneural TFs to induce neuronal differentiation. BMP7, which is secreted from the dorsal telencephalic midline (Furuta et al., 1997) induces Id1 or Id2 expression in spinal cord and cortical NPCs (Vinals et al., 2004; Le Dreau et al., 2018). Id proteins inhibit proneural gene function by sequestering E proteins to prevent their heterodimerization with bHLH TFs (Le Dreau et al., 2018). Furthermore, Id1 induced by BMP4 promotes Ascl1 protein degradation to prevent this TF from promoting neuronal differentiation (Vinals et al., 2004).

Another important aspect of astrocyte differentiation is the timing of when NPCs switch from neurogenesis to gliogenesis. Cytokines are critical regulators of this switch (Barnabe-Heider et al., 2005), but the proneural genes are also involved, as gliogenesis occurs precociously in *Neurog2^–/–^;Ascl1^–/–^* cortices (Nieto et al., 2001). Notably, a similar precocious differentiation of glial cells is seen in *Neurod4^–/–^;Ascl1^–/–^* cortices in the tectum, hindbrain and spinal cord (Tomita et al., 2000), suggesting similar processes may be at play in other brain regions. One interpretation of these data is that in the absence of two proneural genes, neurogenesis cannot take place and instead, gliogenesis ensues. Another interpretation is that *Neurog2* and *Ascl1* regulate temporal identity transitions through the co-dependent activation of a unique set of downstream genes. Consistent with the latter interpretation, *Neurog2* and *Ascl1* are also together required to regulate the timing of cortical neurogenesis, as evidenced by the precocious differentiation of supragranular neurons (Dennis et al., 2017) in *Neurog2^–/–^;Ascl1^–/–^* cortices (Dennis et al., 2017). Mechanistically, Neurog2 and Ascl1 regulate the timing of cortical neurogenesis as both proteins are required to transactivate Fezf2, a critical component of the de-repression circuit that specifies laminar identities. How these genes regulate the timing of cortical gliogenesis is less clear. A simple competitive model may explain these findings, as highlighted above, with the loss of Neurog2 and Ascl1 preventing the sequestration of transcriptional co-activators away from Stat1/3 and Smad1 TFs. However, the recent identification of a slew of transcriptional targets that are co-bound and co-regulated by Neurog2 and Ascl1 in cortical NPCs may shed new light into this process (Han et al., 2020).

## Regulation of Proneural Gene Function at the Translational and Post-Translational Level

Several studies suggest that *Neurog2* and *Ascl1* fate specification activities are temporally regulated. For example, *Neurog2* is only necessary and sufficient to specify a glutamatergic neuronal identity in cortical NPCs before E14.5 (Schuurmans et al., 2004; Li et al., 2012), whereas it promotes NPC progenitor transitions from aRG to INP (Britz et al., 2006) and neuronal migration (Heng et al., 2008) after E14.5. *Ascl1* is also normally expressed in embryonic cortical NPCs (Britz et al., 2006; Han et al., 2020), albeit at lower levels than in subcortical domains, but it does not induce the differentiation of these cells into GABAergic neurons or oligodendrocytes, although it may transactivate oligodendrocyte genes postnatally (Han et al., 2020). Temporally constrained, *Ascl1* is upregulated in *Neurog2*^–/–^ cortical NPCs throughout the neurogenic period but can only respecify these cells to a GABAergic fate before E14.5 (Britz et al., 2006). Temporal changes in *Neurog2* cortical function are not surprising, considering that several differences in early and late cortical NPCs have previously been documented. For example, only early, pre-neurogenic cortices respond to the proliferative activity of Wnts (Viti et al., 2003b) and the ventralizing activity of Shh (Kohtz et al., 1998), while conversely, only late-stage NPCs respond to the gliogenic activity of CNTF (Molne et al., 2000; Takizawa et al., 2001; Viti et al., 2003a; Song and Ghosh, 2004). Several non-mutually exclusive molecular regulatory mechanisms controlling *Neurog2* and *Ascl1* functions in the cortex help explain these confounding findings.

### Regulation of Proneural Gene Translation

**FIGURE 3:**
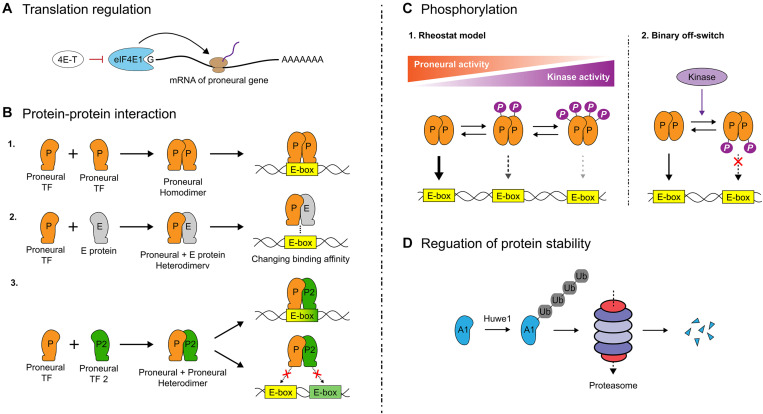
Translational and post-translational regulation of proneural gene function. **(A)** The timing of cortical neurogenesis is controlled by eukaryotic initiation factor 4E1 (eIF4E1) and eIF4E-Binding Protein 4E-T, components of the eukaryotic translational machinery. These factors bind proneural gene transcripts, inhibiting proneural genes translation. **(B)** To bind DNA, bHLH TFs must dimerize. Proneural TF homodimers bind to regions with their cognate E-box sequences. Heterodimers with E proteins may enhance proneural TF binding to DNA if the proneural TF and E protein share a preferred E-box motif; otherwise, DNA binding is impaired. Heterodimerization between proneural TFs similarly may enhance or impede DNA binding depending on the E-box content of a target gene. **(C)** Beyond regulating proneural TF-mediated cell fate choices and neuronal migration, phosphorylation of proneural TFs generally decreases their transcriptional activity. The classical rheostat model holds that progressive phosphorylation of serines or threonines by proline directed serine-threonine kinases (e.g., Cdk, Erk, and Gsk3) at serine-proline (SP) or threonine-proline (TP) sites in proneural TFs decreases their activity depending on the number of sites phosphorylated. More recently, a single conserved residue has been discovered in proneural TFs at the loop/helix-2 junction, which when phosphorylated acts as binary off switch for proneural TF activity, with this mechanism overriding the rheostat mechanism. **(D)** Proneural TFs have short intracellular half-lives and are degraded through the ubiquitin-proteasome pathway. Recently, the E3 ligase Huwe1 was identified as a critical regulator of Ascl1 stability in the adult V-SVZ, with loss of Huwe1 leading to NPC depletion due to sustained Ascl1 expression inducing continuous neuronal differentiation.

There is a tendency to consider the presence of gene transcripts as an indication that a gene is ‘active’ in a particular cell type, but there are many downstream regulatory events that must also be considered. The first consideration is whether transcripts are translated into proteins. Early studies revealed that *Neurog1, Neurog2* and *Ascl1* transcripts are present in many more telencephalic cells than the proteins, but the mechanisms of translational control were not elucidated until recently. A ground-breaking study found that a large host of transcribed neuronal differentiation genes are not translated in the developing cortex (Yang et al., 2014). This study showed that eukaryotic initiation factor 4E1 (eIF4E1) and the eIF4E-Binding Protein, 4E-T, components of the eukaryotic translational machinery, form P-body-like complexes that bind proneural bHLH mRNAs to inhibit their translation, a mechanism of translational control critical for controlling the timing of cortical neurogenesis (Yang et al., 2014) (Figure 3A). Since then, many additional proteins have been identified that control the translation of proneural and neural differentiation genes, including other components of the translational machinery and critical RNA binding proteins (Amadei et al., 2015; Zahr et al., 2018, 2019). Future work will be required to identify specific RNA binding proteins that control the stability and translation of proneural gene transcripts.

### Protein–Protein Interactions

The requirement for dimerization presents numerous opportunities for combinatorial control of bHLH transcriptional activity (Figure 3B). A given bHLH dimer will have its own E-box specificity, and while a comprehensive picture of the differential binding patterns of homo- and heterodimers is not yet known, preferred binding motifs for certain proneural TFs have been discovered in mouse and fish (Seo et al., 2007; Lin et al., 2010; Wapinski et al., 2013; Raposo et al., 2015; Pfurr et al., 2017; Aydin et al., 2019) and certain bHLH dimers are less transcriptionally active than others. As mentioned earlier in this review, Ascl1 preferentially binds to CAGCTG motifs in genomic regulatory regions (Wapinski et al., 2013; Raposo et al., 2015; Aydin et al., 2019), while Neurog1 and Neurog2 preferentially bind CADATG motifs (where D = A/G/T) (Seo et al., 2007; Madelaine and Blader, 2011; Aydin et al., 2019). E47 has been shown to preferentially bind CAGSTG motifs (where S = C/G) (Lin et al., 2010; Pfurr et al., 2017). Recently it has been shown that E proteins alter the neurogenic strength of proneural TFs through physical interactions in a context-specific, E-box-dependent manner, by either synergizing with Ascl1 on CAGSTG motifs or impeding Neurog2’s binding to CADATG motifs (Le Dreau et al., 2018). For example, misexpression of *E47* and *Ascl1* in spinal cord NPCs increases differentiation relative to *Ascl1* alone (Le Dreau et al., 2018), but the opposite effect is observed for co-electroporation of *E47* with *Neurog2*, either in spinal (Le Dreau et al., 2018) or cortical (Li et al., 2012) NPCs. However, there are other regulatory considerations, including that E47 heterodimerization enhances Neurog2 (Li et al., 2012) and Ascl1 (Vinals et al., 2004) protein stability, which can influence their transactivation of some target sites. The finding of E-box-dependent cooperativity of E proteins with proneural TFs leads us to the important consideration that proneural TF activity at downstream targets is modulated by the availability of appropriate dimerization partners. Indeed, in the chick spinal cord, when E protein availability is limited due to its sequestration by Id proteins, Ascl1 proneural strength is negatively impacted due to the reduction in Ascl1∼E47 heterodimers, which effectively transactivate CAGSTG E-box-containing downstream targets (Le Dreau et al., 2018). Conversely, Neurog2 transactivation of CADATG E-box-containing downstream targets is stabilized by a reduction in E47 availability due to Id sequestration (Le Dreau et al., 2018).

Heterodimers can also form between two proneural bHLH TFs. Examples include Neurog1∼Neurog2 heterodimers, which form in E12.5 cortical NPCs (Han et al., 2018) and are likely functionally important, as evidenced by the reduced *Hes5* expression (i.e., Notch signaling) and precocious neurogenesis that occurs in E12.5 *Neurog1*^–/–^ cortices (Schuurmans et al., 2004; Han et al., 2018). Mechanistically, Neurog1∼Neurog2 heterodimers have a reduced capacity to induce neurogenesis compared to Neurog2∼Neurog2 homodimers, leading to the conclusion that Neurog1 slows the pace of cortical neurogenesis at early stages (∼E12.5) when there are higher levels of Neurog1/Neurog2 co-expression (Han et al., 2018). Interestingly, co-expression of *Neurog2* with *Neurod4* accelerates cortical neurogenesis (Mattar et al., 2008), and while protein–protein interactions were not assessed, it remains possible that either Neurog2∼Neurod4 heterodimers have an enhanced capacity to transactivate target genes, or these bHLH TFs form other dimers that bind to distinct E-boxes located in the same regulatory regions of a target gene. Notably, there are other examples of bHLH proneural genes and differentiation genes having co-operative functions, including in the developing retina (Akagi et al., 2004) and hippocampus (Schwab et al., 2000).

Neurog2∼Ascl1 heterodimers have also been identified (Gradwohl et al., 1996; Han et al., 2020), with evidence suggesting that they are non-functional, as predicted by the differential enrichment of bound E-box motifs for each TF identified in ChIP-seq experiments (Aydin et al., 2019). Indeed, co-expression of *Neurog2* and *Ascl1* blocks the transactivation of promoters specific to each TF *in vitro* in transcriptional reporter assays, as well as blocking the ability of *Ascl1* to induce *in vivo* proliferation and Sox9 expression (a glioblast marker in cortical NPCs) as well as the ability of *Neurog2* to induce *in vivo* glutamatergic neuron formation (Han et al., 2020). Despite the inhibitory interactions between Neurog2 and Ascl1, and while most of the genes activated by Neurog2 and Ascl1 do not overlap, there are also some commonly regulated genes (Masserdotti et al., 2015; Aydin et al., 2019). Moreover, the gene regulatory network (GRN) that is associated with Neurog2/Ascl1 double^+^ cortical NPCs is distinct from the GRNs associated with single^+^ NPCs. Neurog2 and Ascl1 could regulate a distinct repertoire of genes when in combination through two potential modes of action. Firstly, they could act on gene regulatory elements that contain both Neurog2- and Ascl1-specific binding sites, as exemplified by *Dll1*, which has two distinct enhancers that are specifically activated by Neurog2 (DeltaN) or Ascl1 (DeltaM) (Castro et al., 2006). Alternatively, enhancers may contain hybrid E-boxes that are bound equally well by Neurog2 and Ascl1, including when they form heterodimers, as exemplified by the *Dll3* promoter (Henke et al., 2009). Further studies on commonly regulated targets of Neurog2 and Ascl1 will aid our understanding of how cortical NPC fate decisions are regulated.

Finally, proneural TFs also form physical interactions with other non-bHLH TFs that play critical roles in cortical development. For example, Neurog2 synergizes with the T-box TF Tbr2, expressed in INPs, to control the radial migration of cortical neurons (Sessa et al., 2017). Neurog2 and Tbr2 control migration by synergistically transactivating *Rnd2*, a critical regulator of cortical neuron migration (Sessa et al., 2017). Other TFs have also been shown to associate with proneural TFs to regulate their functions. For instance, Ascl1 and the POU domain TFs Brn1 and Brn2 cooperatively bind the Dll1 promoter (Castro et al., 2006). Similarly, in the fly, senseless cooperates with atonal to regulate proneural activity (Nolo et al., 2000), and Myt1 is required for optimal Neurog2 proneural activity in Xenopus (Quan et al., 2004). The future identification of additional proneural TF binding partners in cortical NSCs/NPCs will aid in our understanding of the complex GRNs that underlie development of this brain region.

### Phosphorylation of Proneural TFs

Intracellular kinases are key intermediaries between the environment and the cell nucleus, so understanding their impact on proneural TFs can reveal how environmental cues regulate cortical neurogenesis. Interestingly, DNA-binding proteins and TFs are often natively unfolded and intrinsically disordered (Ward et al., 2004), with disordered regions targeted by twice as many kinases as structured domains (Gsponer et al., 2008). Neurog2 is an example of an intrinsically disordered TF that is targeted by various kinases that modulate its activity in a context-dependent manner (McDowell et al., 2014). In general, N- and C-terminal phosphorylation outside of the bHLH domain has inhibitory effects on bHLH proneural activity, but other processes can be promoted by phosphorylation, as highlighted below.

#### Rheostat Model

The rheostat model holds that progressive phosphorylation of TFs results in a graded, finely tuned reduction in DNA binding and hence, transcriptional activity (Pufall et al., 2005) (Figure 3C). This model has garnered support with regards to proneural TFs from experimental work in mouse and xenopus (Ali et al., 2011; Hindley et al., 2012; McDowell et al., 2014; Hardwick and Philpott, 2015). Proneural TFs are phosphorylated by a host of proline-directed serine/threonine (S/T) kinases, including cyclin-dependent kinases (Cdk – on Neurog2 and Neurod4) (Ali et al., 2011; Hindley et al., 2012; McDowell et al., 2014; Hardwick and Philpott, 2015), GSK3 (on Neurog2) (Li et al., 2012) and ERK (on Ascl1) (Li et al., 2014). These S/T kinases can progressively phosphorylate nine serine-proline (SP) sites in Neurog2, six SPs in Ascl1 and a combined seven threonine-proline (TP) and SP sites in Neurod4. In xenopus, the progressive phosphorylation of Neurog2 SP phosphoacceptor sites by Cdks limits its ability to drive neurogenesis, with the number of serine-proline sites phosphorylated more important than their location (McDowell et al., 2014). Based on the “cell cycle length hypothesis,” NPCs that differentiate have a longer G_1_ phase, and the prediction is that Cdk activity would be reduced in these cells so that Neurog2 would be underphosphorylated, thereby in a permissive state to initiate transcription of neurogenesis-associated target genes (Calegari and Huttner, 2003). Conversely, Cdk levels would rise in dividing NPCs, increasing proneural TF phosphorylation, and inhibiting transactivation of downstream gene (Ali et al., 2011). Notably, Cdk inhibits Neurog2-mediated transactivation of *Neurod1*, a neuronal differentiation gene, more robustly than *Dll1*, which induces neighboring NPCs to proliferate (Hindley et al., 2012), suggesting Cdk plays a critical role in regulating neural development. Accordingly, in the developing cortex, the proneural competence of Neurog2 also declines during late neurogenesis due to increasing levels of GSK3-mediated phosphorylation (Li et al., 2012).

#### Cell Fate Choice

Phosphorylation by SP kinases not only controls the decision to proliferate or differentiate, but also influences cell fate choices that are important in normal development but can also impact tumor formation. In the spinal cord, phosphorylation of Neurog2 S231 and S234 (SP sites) promotes the formation of TF complexes between Neurog2 and the adaptor protein Ldb1, which recruits LIM-homeodomain TFs Isl1 and Lhx3 to form a complex that transactivates motor neuron specific genes (Ma et al., 2008). Similarly, in the embryonic telencephalon, intermediate vs high RAS/ERK activation levels dictate whether *Ascl1* selects GABA vs OPC transcriptional targets, respectively (Li et al., 2014) (Figure 2B). Notably, there is also a correlation between higher pERK levels and more glial cells in pilocytic astrocytomas, compared to lower levels of pERK and fewer glial cells in ganglioglioma, despite these two tumor types sharing the same bRAFv600e mutation (Li et al., 2014). It is interesting to speculate that ERK-mediated phosphorylation of ASCL1 controls, at least in part, the different cellular features of these genetically similar tumors. Similarly, the tumorigenicity of bHLH TF Olig2 is driven by its phosphorylation status, with phosphomimetic mutations rendering it more tumorigenic, and phospho-dead mutations non-tumorigenic (Sun et al., 2011). In line with this, phosphorylation of a conserved triple serine motif in Olig2 promotes its unorthodox ‘antineural’ pro-proliferative functions, instead of the ‘proneural-like’ activity of inducing an oligodendrocyte fate (Sun et al., 2011). These data highlight the importance of phosphorylation events of bHLH TFs not only for normal development, but also in tumorigenesis.

#### Binary ‘Off’ Switch

A single conserved S/T residue at the Loop/Helix 2 (L-H2) junction acts as an evolutionarily conserved, binary ‘off’ switch for both vertebrate and invertebrate proneural TFs (Quan et al., 2016) (Figure 3C). 3D modeling revealed that the conserved S/T residue faces the DNA backbone such that addition of a negatively charged phosphate group would generate electrostatic repulsion between the TF and DNA, effectively rendering the TF a null mutant. At the L-H2 junction, Drosophila ato and vertebrate Atoh1 are phosphorylated on S292 by protein kinase A (PKA), while Neurog2 is phosphorylated on T149 by MARK1 and PLK1. A phosphomimetic mutation (T149D) destabilized Neurog2 binding to DNA and abolished its ability to induce neurogenesis in cortical NPCs *in vivo* (Quan et al., 2016). Strikingly, this binary off-switch essentially ‘trumps’ the rheostat model of control, as introduction of a single phosphomimetic mutation in the conserved L-H2 region of Ascl1 and Neurog2 prevents their proneural activities, even when ‘activating’ phospho-null mutations are introduced in SP and TP sites throughout the proteins (Hardwick and Philpott, 2018a, b). The speculation that these different regulatory modes may come into play at different developmental time points depending on an NPC’s ‘kinase environment’ is interesting due to the possibility of rapidly halting proneural activity to ensure that correct neuronal numbers are generated (Hardwick and Philpott, 2018a).

#### Neuronal Migration

Neurog2 is also phosphorylated on Tyr241, a residue outside the bHLH domain that is dispensable for proneural activity, but required to specify a polarized neuronal phenotype and establish appropriate radial migration patterns (Hand et al., 2005). Mutation of Y241 leads to defects in neuronal migration and neuronal morphogenesis defects in the neocortex (Hand et al., 2005), in part by preventing the association between Neurog2 and CBP, a transcriptional co-activator protein that is required for Neurog2 to transactivate genes that control neuronal migration and dendritic polarity, such as Dcx (Ge et al., 2006). Notably, the ability of Neurog2 to sequester CBP is also proposed to be important for the indirect repression of RhoA, which must be downregulated for cortical neurons to migrate appropriately (Ge et al., 2006).

### Regulation of Proneural TF Protein Stability

It is now well established that both fly (Kiparaki et al., 2015) and vertebrate (Nguyen et al., 2006; Ali et al., 2011; Li et al., 2012; Kovach et al., 2013) proneural TFs have very short intracellular half-lives (∼20–40 min). In the fly, two destabilizing motifs were found in the proneural TF encoded by *scute* (Sc); the transactivation domain (TAD) and an SPTSS motif, including a phosphoacceptor site for proline-directed S/T kinases (Kiparaki et al., 2015). Notably, S-A mutations in the SPTSS motif dramatically stabilized fly Sc (Kiparaki et al., 2015), and similarly, there was an ∼2-fold increase in Neurog2 stability when all 9 SP sites were mutated to SA in Xenopus (Ali et al., 2011). Removal of the C-terminal TAD domain also dramatically stabilizes murine Neurog2 (Li et al., 2012) and fly Sc (Kiparaki et al., 2015) proteins. However, while the forced tethering of mouse Neurog2 to E47 (Li et al., 2012), or E12 to human ASCL1 (Sriuranpong et al., 2002) stabilizes these proneural TFs, Sc heterodimerization with fly Daughterless (Da), the E-protein homolog, promotes further degradation (Kiparaki et al., 2015). Thus, there are critical differences in how proneural protein stability is regulated, but nevertheless, in all species, proneural TFs have short intracellular half-lives.

There is growing evidence that proneural protein degradation is mediated by the ubiquitin-proteasome degradation system (UPS) (Figure 3D). Ubiquitin moieties form isopeptide bonds with lysine residues in substrate proteins that are targeted for degradation through the actions of three enzymes; ubiquitin-activating (E1), ubiquitin-conjugating (E2), and ubiquitin ligase (E3) enzymes (Komander and Rape, 2012). Polyubiquitylated substrates undergo degradation through the UPS (Johnson et al., 1995). Proneural TFs regulated by UPS include murine (Vinals et al., 2004) and human (Sriuranpong et al., 2002) Ascl1, xenopus Neurog2 (Vosper et al., 2007, 2009) and the fly protein Sc (Kiparaki et al., 2015). Recently, the E3-ligase Huwe1 (HECT, UBA and WWE domain containing 1) was identified as a critical destabilizer of Ascl1 in the postnatal hippocampus (Urban et al., 2016) (Figure 3D). Huwe1 maintains adult NSCs in quiescence by targeting Ascl1 for degradation, with excess NSCs entering the cell cycle upon conditional *Huwe1* deletion, resulting in a depletion of the NSC pool (Urban et al., 2016). Spatial resolution is also emerging in the picture of Ascl1 regulation by Huwe1 (Gillotin et al., 2018). Cytoplasmic Ascl1 is predominantly attached to longer polyubiquitin chains on lysines within the bHLH region and is rapidly targeted for degradation by the UPS, while chromatin-bound Ascl1 is ubiquitylated with shorter chains on N-terminal and bHLH lysines but is not targeted for degradation (Gillotin et al., 2018).

While comparable E3-ligases have yet to be identified for Neurog1 and Neurog2 in the cortex, Fbxo9 is an E3-ligase that destabilizes Neurog2 in the developing dorsal root ganglia (Liu et al., 2020). Mechanistically, in Xenopus, Neurog2 is stabilized by Cdk inhibitor p27Xic1 (Nguyen et al., 2006), and p27Xic1 promotes neurogenesis partially due to its stabilizing effect on Neurog2 (Vernon et al., 2003), but whether there is a direct involvement with UPS is not known.

## Epigenetic Regulation of Proneural bHLH Activity

### Temporal and Spatial Restrictions on Proneural Gene Function

Transcription factors that act as cell fate determinants generally transactivate lineage-specific target genes only in certain cellular contexts (Gascon et al., 2017). For instance, the glutamatergic neuronal fate-specifying properties of *Neurog2* are temporally restricted; in the embryonic cortex, *Neurog2* only efficiently induces neurogenesis before E14.5 (Li et al., 2012). Regional restrictions also occur, with *Neurog2* efficiently able to induce neurogenesis in the dorsal and not ventral telencephalon (Mattar et al., 2008). Proneural genes have also emerged as critical architects of neuronal reprogramming (Wilkinson et al., 2013). However, in keeping with their tight contextual regulation, they are not active in all cell types; *Ascl1* is a potent neuronal reprogramming factor in fibroblasts (Vierbuchen et al., 2010; Caiazzo et al., 2011; Kim et al., 2011; Pang et al., 2011; Pfisterer et al., 2011; Son et al., 2011), hepatocytes (Marro et al., 2011), cardiomyocytes (Chuang et al., 2017), astrocytes (Rivetti di Val Cervo et al., 2017) and pluripotent stem cells (Yang et al., 2017), and not in the adult neocortex (Grande et al., 2013), hippocampus or spinal cord (Ohori et al., 2006; Jessberger et al., 2008). Conversely, *Neurog2* has a more limited ability to convert astrocytes to neurons (Grande et al., 2013; Gascon et al., 2016, 2017; Russo et al., 2020; Stricker and Gotz, 2020) and is used less often for neuronal reprogramming as it must be combined with other signals to become a potent lineage converter (Gascon et al., 2016; Russo et al., 2020).

Understanding how the lineage determination activities of *Neurog2* and *Ascl1* are restricted requires an understanding of how they interact with factors that remodel chromatin. In the field of cellular reprogramming, it is widely held that epigenetic regulators act as ‘gatekeepers’ to prevent cells from transiting from one cell fate to another other organisms, controlling genome accessibility to lineage-specifying TFs (Tursun et al., 2011; Cheloufi et al., 2015; Gascon et al., 2017). Notably, chromatin structure, and hence the accessibility of promoters/enhancers, changes during cortical development (Kishi et al., 2012). Moreover, even within the cortical NPC pool at a single age, there are distinct NPC populations defined as *Neurog2/Ascl1* negative, single^+^ or double^+^ NPCs that each have distinct chromatin landscapes (Han et al., 2020). Below we review how proneural bHLH TFs intersect with chromatin modifiers to influence the genome architecture, ultimately affecting their ability to bind and transactivate target genes.

### Epigenetic and Metabolic Regulation of Proneural TF Function

**FIGURE 4:**
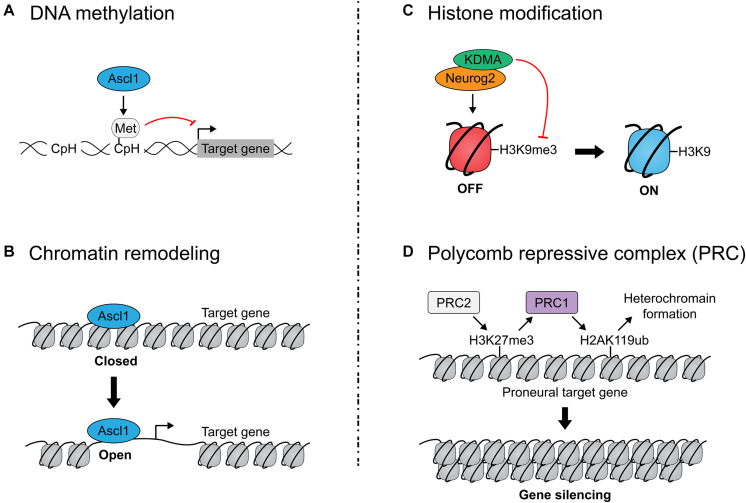
Epigenetic regulation of proneural transcription factor activity. **(A)** Ascl1 induces non-CpG (CpH) methylation of fibroblast-specific genes during reprogramming of mouse embryonic fibroblasts to neurons. DNA methylation represses gene expression by compacting the genome and rendering it inaccessible. **(B)** Ascl1 is a pioneer factor which can bind nucleosome-bound DNA (in a ‘closed’ state) and facilitate the de-compacting of these sites to permit gene expression. Neurog2 has also been identified as a pioneer factor. **(C)** Neurog2 interacts with the H3K9 demethylase to open chromatin and promote neurogenesis. **(D)** Polycomb repressive complexes (PRC) close chromatin to render target genes inaccessible to proneural TFs. PRC1 ubiquitylates K119 in Histone H2A, while PRC2 methylates Lys27 in Histone H3, altogether leading to heterochromatin formation. Mutation of PRC1/2 genes prolongs proneural TF expression, implicating PRC1/2 in the temporal regulation of proneural TF activity.

Ascl1- and Neurog2 form homo- or hetero-dimers with other bHLH proteins to bind specific E-box motifs in the genome. Neurog2 and Ascl1 are termed ‘pioneer factors’ based on their ability to bind ‘closed’ (nucleosome-bound) chromatin and facilitate the opening of these sites for TF binding (Wapinski et al., 2013; Aydin et al., 2019) (Figure 4A). leading to the opening of distinct chromatin regions which render downstream differentiation genes accessible (Aydin et al., 2019). Several studies have begun to unravel how Neurog2 and Ascl1 influence the chromatin landscape through interactions with different epigenetic modifiers and pathways, as summarized below:

#### DNA Methylation

DNA hypermethylation of cytosine residues in CpG sequences, which are typically found in promoters/enhancers, represses gene expression by compacting the genome and rendering it inaccessible to TF binding. By changing DNA methylation patterns, chromatin accessibility is altered, as are downstream gene expression patterns. Notably, DNA can also be methylated on non-CpG sequences (CpH) that are prevalent throughout the genome, including in non-regulatory regions, but these modifications similarly repress gene expression (Guo et al., 2014). Strikingly, in a direct neuronal reprogramming study in which *Ascl1* was overexpressed in mouse embryonic fibroblasts (MEFs), Ascl1 induced CpH methylation of fibroblast-specific genes, but cooperation with other TFs (Brn2, Myt1l) (Wapinski et al., 2013), which increase reprogramming efficiency, was required for methylation patterns to faithfully recapitulate those seen in cortical neurons (Luo et al., 2019) (Figure 4B).

#### Chromatin Remodeling

Ascl1-mediated *trans*-differentiation of MEFs to neurons showed that Ascl1 induces widespread chromatin remodeling (Wapinski et al., 2017). During this switch, there is substantial Ascl1-driven genome-wide remodeling of chromatin architecture near Ascl1 binding sites, leading to a stabilized nucleosome configuration at day 5 which facilitates the stable expression of mature neuronal genes. Notably, the presence of a swift and concerted chromatin switch in this *trans*-differentiation protocol contrasts with the classical ‘step-wise’ view of *in vivo* neuronal development and iPSC reprogramming, emphasizing the limitations of using direct somatic cell reprogramming to model development. In addition, Neurog1 interacts with Brg1, a SWI/SNF chromatin remodeler to aid neurogenesis (Seo et al., 2005).

#### Selective Transactivation of Bound E-Boxes

bHLH TFs act as lineage determinants in multiple tissues, and include Myod1, a master regulator of a skeletal muscle fate (Lee et al., 2020). Strikingly, when misexpressed in MEFs, Ascl1 and Myod1 bind very similar target genes, but to a different degree, and can only induce chromatin opening of sites involved in neuronal and muscle lineage reprogramming, respectively (Lee et al., 2020). However, when Myod1 is overexpressed with Myt1l, which inhibits the acquisition of a muscle identity, Myod1 can induce neuronal differentiation (Lee et al., 2020).

#### Co-activator and Co-repressor Interactions

A common property of TFs that act as transactivators is their association with co-activator proteins that function as histone acetyl transferases (HATs), as exemplified by p300/CBP (Sheikh, 2014). p300/CBP preferentially acetylates lysine residues (K) on histone H3/H4 tails (e.g., H3K27ac), and ‘opens’ chromatin by electrostatic repulsion between negatively charged acetyl groups and DNA. Conversely, histone deacetylases (HDACs) remove these acetyl groups and compact the chromatin. Several papers have documented associations between the proneural bHLH genes and HAT co-activators (Koyano-Nakagawa et al., 1999; Sun et al., 2001; Vojtek et al., 2003; Seo et al., 2005, 2007; Ge et al., 2006). The proneural genes are also thought to indirectly repress gliogenesis by sequestering HAT proteins away from gliogenic genes (Sun et al., 2001; Ge et al., 2006). Interestingly, the bHLH protein Hes1 switches from binding a TLE-HDAC co-repressor complex to HAT binding as neurogenesis proceeds (Ju et al., 2004), highlighting the importance of dynamic interactions between these factors in regulating the timing of neurogenesis.

#### Histone Methylation

Opening of the chromatin to provide access to TF binding is also associated with methylation of H3 histone tails, but here the specific lysine (K) residues are critical. For instance, while RNAPol II and trimethylation (me3) of histone H3K4 cluster at transcription start sites of actively transcribed genes, and histone H3K36me3 is in the body of actively transcribed genes, other chromatin marks are found in silenced regions of the genome (H3K9me3, H3K27me3) (Ringrose et al., 2004; Bernstein et al., 2006; Pavri et al., 2006; Sims and Reinberg, 2006; Muller and Verrijzer, 2009; Tavares et al., 2012). Notably, Neurog2 forms a complex with KDMA, an H3K9 demethylase, to open chromatin and promote neurogenesis (Lin et al., 2017) (Figure 4C). Additionally, Tbr2 physically interacts with JMJD3 histone demethylase, upregulating neuronal-specific genes when co-expressed, potentially by directing JMJD3 to remove repressive H3K27me3 marks (Sessa et al., 2017). Interestingly, Neurog2 physically associates with and shares a majority of its bound genomic target sequences with Tbr2 and acts synergistically on shared target genes in equimolar quantities, so perhaps Neurog2 interacts with and directs JMJD3 activity as well (Sessa et al., 2017).

#### Polycomb Group Proteins

Only a handful of epigenetic gatekeepers are known; most influence chromatin structure, some via interactions with Polycomb group (PcG) proteins, which close chromatin (Tursun et al., 2011; Cheloufi et al., 2015; Gascon et al., 2017). PcG proteins modify chromatin to confer transcriptional repression and exist in two repressive complexes: PRC1 (Ring1a, Ring1b, etc.) and PRC2 (Eed, Suz12, Ezh1/2, etc.). PRC1 catalyzes the ubiquitylation of K119 in Histone H2A (H2AK119ub), while PRC2 catalyzes the methylation of Lys27 in Histone H3 (H3K27me3), altogether leading to downregulation of nearby genes (Figure 4D).

**FIGURE 5:**
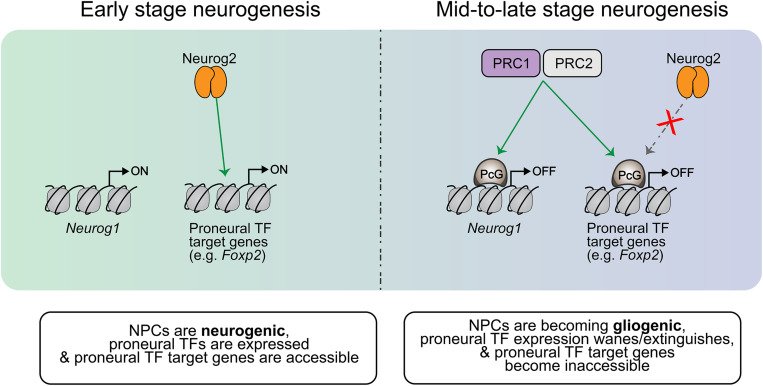
Temporal regulation of proneural gene expression and target gene availability. In early NPCs, *Neurog1* expression is turned on and proneural TF target genes are available (not repressed). As the cortical neurogenic period progresses, PRC1/2 catalyze the addition of PcG proteins to repress certain genes which are part of the neuronal differentiation cascade. For example, PRC1/2 repress *Foxp2* in mid-neurogenesis, making it inaccessible to Neurog2 and thereby terminating the Neurog2-mediated specification of corticothalamic neurons past this period. This PRC1/2 activity thus controls the temporal competence of NPCs, dictating the differentiation programs that may be initiated at different stages of development. PRC1/2 also extinguish the expression of proneural TFs like *Neurog1* later on in the neurogenic period, and this contributes to the induction of gliogenic differentiation programs within NPCs as Neurog1 has been shown to prevent the induction of such programs.

PRC1 and PRC2 control temporal NPC fate competence by regulating proneural gene expression and proneural TF target gene availability (Figure 5). PRC1 controls the temporal window of *Neurog1*, but not *Neurog2*, expression, with mutation of *Ring1b* extending *Neurog1* expression into late-stage neurogenesis (e.g., E17) (Hirabayashi et al., 2009). Extending *Neurog1* gene expression and derepressing the expression of neuronal lineage genes, *Ring1B* and *Ezh2* deletions also delayed the onset of gliogenesis, indicating that PRC1 and PRC2 control the neurogenic-to-gliogenic fate switch in cortical NPCs (Hirabayashi et al., 2009). Recently, Ring1b was also shown to control the spatial expression pattern of *Neurog1*, with *Ring1b* deletion expanding *Neurog1* expression further ventrally to overlap with *Ascl1* expression in the murine E10 telencephalon (Eto et al., 2020). Surprisingly, while Neurog1/2 have been shown to repress *Ascl1* expression in the cortex, establishing mutually exclusive expression patterns, *Ring1b* deletion markedly increased *Neurog1/Ascl1* double^+^ NPCs, suggesting that PcG proteins may also regulate mutual exclusivity of proneural TF expression (Eto et al., 2020). Ring1b has also been shown to temporally limit the production of subcerebral neurons by NPCs through repression of *Fezf2* expression (Morimoto-Suzki et al., 2014), which is a known transcriptional target of Neurog2. Furthermore, PRC2 components *Eed* and *Suz12* and PRC1 components *Ring1a* and *Ring1b* suppress the ability of Neurog2 to induce a corticothalamic differentiation program by repressing downstream target, Foxp2 (Oishi et al., 2020). In this way, PRC1 and PRC2 progressively regulate targets of proneural TF-induced differentiation programs to control NPC temporal fate competence.

Recent RNA-Seq evidence from FlashTag-isolated apical NPCs further substantiates the claim that PRC2 temporally regulates NPC competence, with mutation of *Eed* (inactivation of PRC2) leading to precocious neurogenesis (Telley et al., 2019). *Eed* mutants further displayed precocious generation of typically late-born neurons and increased cell cycle exit in early neurogenesis, leading to a terminal decrease in cortical thickness (which indicates a shorter neurogenic period) (Telley et al., 2019). Similarly, the PRC2 catalytic component Ezh2 was shown to control developmental rate, with loss of Ezh2 shifting NPCs toward differentiation over proliferation as well as an earlier induction of gliogenesis (Pereira et al., 2010).

Overall, these findings implicate PcG proteins as crucial temporal regulators of proneural transcriptional programs, operating to limit the timeframe of subtype specification by proneural TFs by occluding proneural TF target genes. These critical epigenetic regulators do not act alone, though; temporal NPC fate specification is simultaneously and robustly regulated by other mechanisms such as declining proneural transcriptional activity.

### Proneural TFs Are Regulated by Metabolism

The global metabolic changes associated with neuronal differentiation have only recently begun to be elucidated (Agostini et al., 2016), and new work is emerging investigating the metabolic regulators of Neurog2- or Ascl1- driven direct neuronal conversion strategies in both human and mouse cells. As we have expounded in this review, proneural TF function is context dependent, and thus the efficiency at which proneural TFs induce neuronal conversion must also be contingent on the bio-energetic differences of starting cell types in conversion protocols. Indeed, it has been shown that oxidative stress (Gascon et al., 2016) and fatty acid B-oxidation (Russo et al., 2020) impose major hurdles in the reprogramming of astrocytes, which rely on glycolytic metabolism (McKay et al., 1983; Tsacopoulos and Magistretti, 1996), to neurons, which rely on oxidative metabolism (Herrero-Mendez et al., 2009). Thus, genetic or pharmacological manipulations to reduce oxidative stress, such as Bcl-2 overexpression or the addition of forskolin, vitamin E, and calcitriol, increases the efficiency (both the speed and number of converted cells) of proneural TF-driven neuronal conversion (Gascon et al., 2016). Bcl-2 functions independently of its canonical anti-apoptotic role to reduce reactive oxygen species (ROS) and facilitate more efficient proneural TF-driven fate transitions; hence, co-transduction with Ascl1 or Neurog2 significantly improves astrocyte-to-neuron conversion efficiencies and neuronal maturation *in vitro*, as well as *in vivo* in injured mouse cortex (Gascon et al., 2016). Interestingly, microarray analysis of *Ascl1*-transduced MEFs revealed that forskolin treatment enriched BMP and Wnt signaling pathway genes (Gascon et al., 2016), both of which influence proneural TF expression, though it is unknown if the reprogramming enhancement seen in this study is caused by direct effects on proneural TF function or by creating a more permissive cellular environment for reprogramming to occur. Further underscoring the importance of bioenergetics in the regulation of proneural TF function, one-fifth of the mitochondrial proteome differs between astrocytes and cortical neurons, and CRISPRa-mediated induction of neuronal, but not astrocytic, mitochondrial proteins enhances the efficiency of Ascl1-driven reprogramming (Russo et al., 2020). The most robust enhancement of Ascl1-driven reprogramming occurs with co-transduction of neuronal-specific antioxidant protein Sod1, leading to increases in recruitment of cells for reprogramming, speed of conversion into neurons, and lifespan of converted neurons (Russo et al., 2020). These new findings add an important layer of complexity to the web of proneural TF regulation, and when considered together with the other regulatory mechanisms detailed in this review, will allow for the design of more efficient proneural TF-driven neuronal reprogramming protocols in the future.

## Discussion

Proneural TFs are critical regulators of neural cell differentiation and subtype specification, contributing to the enormous cellular diversity observed in the cortex. It therefore holds that proneural TFs are themselves tightly regulated, and these interplaying mechanisms of regulation are being elucidated by the scientific community. A coherent picture of robust regulation is emerging, one with overlapping mechanisms that limit proneural TF actions to certain temporal windows in development. Altogether, the current literature suggests a temporal sequence of regulation as follows. Initially, proneural gene transcript expression is induced by early morphogenetic signals and modulated in undifferentiated NPCs by antagonistic Notch signaling and by other cross-repressive proneural TFs. In NPCs where proneural gene transcript expression is permitted, translation may still be prevented by repressive eIF4E-4E-T complexes. Once proneural gene expression becomes sustained in Notch(-) NPCs, proneural TFs may then be translated into TFs which are regulated in their stability and transcriptional activity by different dimerization partners and by phosphorylation of different residues within their bHLH, N- and C-terminal domains. When the actions of a proneural TF are no longer needed, the protein may be degraded proteolytically by polyubiquitylation (for which exact enzymatic candidates are emerging), or by broader inhibition at the epigenetic level (with mechanisms like repressive PcG proteins limiting proneural TF access to downstream targets).

In the future, the ultimate application of the multifaceted picture of proneural gene regulation that we have painted in this review would aid in the refinement and improvement of neuronal reprogramming strategies incorporating these proneural genes for regenerative medicine purposes.

## Author Contributions

A-MO and CS: conceptualization. A-MO, SH, and CS: writing—review and editing. A-MO and SH: artwork. CS: funding acquisition. All authors contributed to the article and approved the submitted version.

## Conflict of Interest

The authors declare that the research was conducted in the absence of any commercial or financial relationships that could be construed as a potential conflict of interest.
